# Neuroprotective effect of Danshensu derivatives as anti-ischaemia agents on SH-SY5Y cells and rat brain

**DOI:** 10.1042/BSR20130032

**Published:** 2013-08-30

**Authors:** Sunisa Seetapun, Jia Yaoling, Yang Wang, Yi Zhun Zhu

**Affiliations:** *Department of Pharmacology, School of Pharmacy, Fudan University, 826 Zhangheng Road, Shanghai 201203, People's Republic of China; †Department of Medicinal Chemistry, School of Pharmacy, Fudan University, 826 Zhangheng Road, Shanghai 201203, People's Republic of China

**Keywords:** anti-apoptosis, anti-oxidative stress, Danshensu derivatives, ischemic stroke, middle cerebral artery occlusion (MCAO), neuroprotective effect, DHE, dihydroethidium, DIPEA, diisopropylethylamine, DMEM, Dulbecco’s modified Eagle’s medium, EDC, 1-(3-dimethylaminopropyl)-3-ehylcarbodiimide hydrochloride, GAPDH, glyceraldehyde-3-phosphate dehydrogenase, GPx, glutathione peroxidase, HOBt, 1-hydroxybenzotriazole, LDH, lactate dehydrogenase, MCAO, middle cerebral artery occlusion, MDA, malondialdehyde, MTT, 3-(4,5-dimethylthiazol-2-yl)-2,5-diphenyl-2*H*-tetrazolium bromide, NAC, *N*-acetyl-L-cysteine, NBS, neuronal bovine serum, ROS, reactive oxygen species, SOD, superoxide dismutase, TTC, 2,3,5-triphenyltetrazolium chloride

## Abstract

Novel Danshensu derivatives (**3–8**) were designed and synthesized to improve bioactivity based on the strategy of ‘medicinal chemical hybridization’. Our previous studies indicated that these compounds exhibited noticeable cardioprotective activities. Here, we investigate whether these novel Danshensu derivatives exert neuroprotective activities. An *in vitro* study revealed that these compounds could increase cell viability and reduce LDH (lactate dehydrogenase) leakage. Moreover, Danshensu-cysteine derivative compounds **6** and **8** could significantly inhibit lipid peroxidation of cell membrane and regulate the expression of apoptosis-related protein (Bcl-2, Bax and caspase 3). An *in vivo* study demonstrated that treatment with compound **6** at 30 mg/kg markedly decreased the infarct volume of MCAO (middle cerebral artery occlusion) insulted rat brain. Furthermore, treatment with compound **6** showed the antioxidant capacity by increasing the activity of SOD (superoxide dismutase) and GPx (glutathione peroxidase) and decreasing the level of MDA (malondialdehyde) and the ROS (reactive oxygen species) production significantly. These results suggested that these novel conjugates exert significant neuroprotective effects as anti-ischaemia agents and those with high potential merit further investigation.

## INTRODUCTION

Ischaemic stroke is one of the major causes of morbidity and mortality worldwide [1,2]. It results from a blockage of blood flow to the region of brain. Ineffective therapies result in the number of poor prognosis in stroke patients. Thus, many studies for stroke have been established in order to understand the pathophysiology and achieve a better therapy. The studies demonstrated the important role of oxidative stress and apoptosis in the progression of neuronal loss and cerebral ischaemia injury [3–5]. Therefore, a search for novel pharmacological agents has been initiated targeting better therapy.

**Scheme 1:**
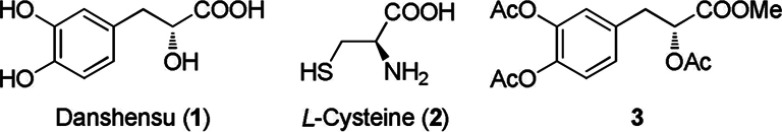
The structures of Danshensu (1), L-cysteine (2) and Danshensu derivatives (3)

**Scheme 2:**
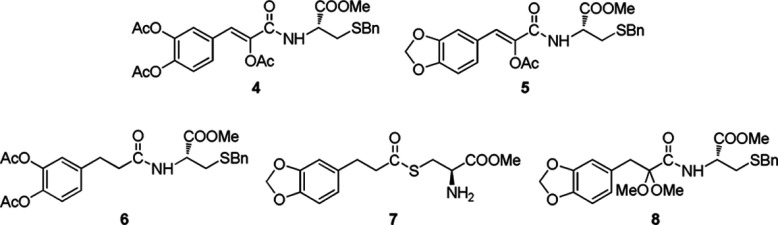
The structures of the conjugated derivatives (4–8)

Danshensu, (*R*)-(+)-3-(3,4-dihydroxyphenyl)-2-hydroxypropanoic acid (**1**, Scheme 1) is the major hydrosoluble active compound of Danshen (Salviae Miltiorrhizae). It has been widely used for therapeutic purpose for decades [6]. Its potent pharmacological effects have been long discovered such as anti-oxidant activity, anti-coagulant, anti-inflammatory, cardioprotective effect, vasodilation and vascular reconstruction [7,8]. In our previous work [9,10], a series of Danshensu and Danshensu-cysteine derivatives were synthesized to improve the biological activity. Moreover, the preliminary pharmacological studies showed that they exhibit significant activity for cardiovascular protection by blocking oxidative stress and apoptosis pathway. Danshensu derivative **3** (Scheme 1) exhibits potent protective activities against hypoxia-induced myocardial cell damage. The structure of the phenolic hydroxyls esterized of the compound enhances the stability and liposolubility as well as easily hydrolysed and readily release bioactive [10]. Therefore, it is highly desirable for novel Danshensu derivatives with more effective pharmacological activity. Recently, the novel amide and thioester conjugates of Danshensu (**1**) and L-cysteine (**2**) derivatives, i.e. compounds **4–7** (Scheme 2), have been synthesized and shown the cardiovascular protection on H_2_O_2_-induced myocardial cell injury [9]. In the present study, we investigated the neuroprotective effect of Danshensu derivative compounds **3–7** as well as novel Danshensu derivative compound **8** on H_2_O_2_-induced neuronal cell injury and a cerebral ischaemic stroke model in rat. Furthermore, the underlying mechanism has been studied preliminarily.

## MATERIALS AND METHODS

### Materials and reagents

DMEM (Dulbecco's modified Eagle's medium) and NBS (neuronal bovine serum) were obtained from Gibco-BRL. H_2_O_2_, NAC (*N*-acetyl-L-cysteine), MTT 3-(4,5-dimethylthiazol-2-yl)-2, 5-diphenyl-2*H*-tetrazolium bromide) and DMSO were purchased from Sigma Chemical. Anti-Bcl-2, anti-Bax and GAPDH (glyceraldehyde-3-phosphate dehydrogenase) were purchased from Proteintech Group Inc.; anti-caspase 3 was obtained from Cell Signaling Technology Inc.; anti-SOD-2 (superoxide dismutase-2) was purchased from Santa Cruz Biotechnology Inc. BCA protein assay kit (Biocolor Bioscience & Technology Company). LDH (lactate dehydrogenase), GSH, MDA (malondialdehyde), SOD and GPx (glutathione peroxidase) kit were purchased from Jiancheng Bioengineering Institute. Hoechst 33258 staining, Tissue Mitochondria Isolation Kit and RIPA lysis buffer were purchased from Beyotime Bioengineering Institute. All compound (except for NAC) solutions in DMSO were freshly prepared. Final DMSO concentration in media did not exceed 0.05% and did not affect cell viability.

### Chemical synthesis of Danshensu–cysteine conjugates

**Scheme 3:**
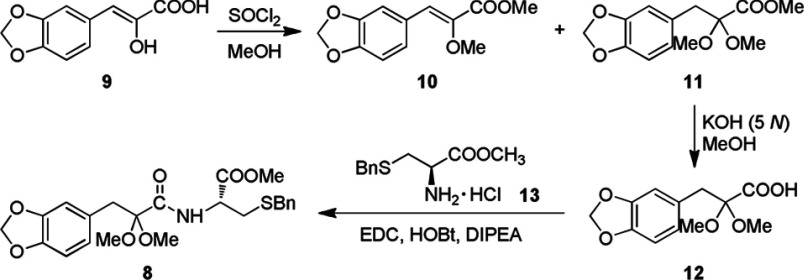
The synthesis of target conjugate 8

Based on the ‘medicinal chemical hybridization strategy’, which has been widely used to design a polyvalent drug, the target conjugates of 2-acetoxy caffeic acid–cysteine derivatives **4–8** were designed via amidation and thioesterification to enhance bioactivity through synergic effect (Scheme 2). Compounds **4–7** were synthesized according to the procedure reported in our previous work [9]. The conjugate **8** was synthesized as outlined in Scheme 3 *via* the starting material **9**, which could be facilely attained in 62% yield by the condensation of 3,4-methylenedioxybenzaldehyde with *N*-acetylglycine followed by hydrolysis in hydrochloric acid [9]. The protection of hydroxyl and carboxyl groups in compound **9** was carried out in the presence of SOCl_2_ in methanol, resulting in the esterified etherified product **10** and esterified ketal **11** in 21% and 51% yields, respectively. The hydrolysis of ester **11** catalysed by aqueous KOH (5 N) in methanol gave carboxylic acid **12** in 94% yield. Finally, acid **12** was condensed with L-cysteine derivative **13** in the presence of EDC [1-(3-dimethylaminopropyl)-3-ehylcarbodiimide hydrochloride], HOBt (1-hydroxybenzotriazole) and DIPEA (diisopropylethylamine) to provide amide conjugate **8** in 70% yield.

### (*Z*)-Methyl 3-(3,4-methylenedioxyphenyl)-2-methoxycarylate (10) and methyl 3-(3,4-methyle-nedioxyphenyl)-2,2-dimethoxypropanoate (11)

SOCl_2_ (0.3 ml, 0.49 g, 4.1 mmol) was added drop wise into a solution of (*Z*)-3-(3,4-methylenedioxyphenyl)-2-hydroxyacrylic acid (**9**) (0.5 g, 2.4 mmol) in methanol (6 ml) in ice bath for 0.5 h and then the mixture was stirred at room temperature (25°C) for 8 h. The mixture was purified by silica gel column chromatography (eluent: PE-EtOAc, 10:1–5:1), providing compounds **10** (0.12 g, 21%) and **11** (0.32 g, 51%) as a white solid and a yellow oil, respectively. **10**: ^1^H NMR (400 MHz, CDCl_3_) δ 3.73 (s, 3H, –OCH_3_), 3.84 (s, 3H, –COOCH_3_), 6.01 (s, 2H, –OCH_2_O–), 6.81 (d, *J*=8.2 Hz, 1H, 5′-H), 6.92 (s, 1H, –CH=), 7.15 (d, *J*=8.2 Hz, 1H, 6′-H), 7.44 (s, 1H, 2′-H). **11**: ^1^H NMR (400 MHz, CDCl_3_), δ 3.08 (s, 2H, –CH_2_–), 3.36 (s, 6H, (–OCH_3_)_2_), 3.65 (s, 3H, –COOCH_3_), 5.90 (s, 2H, –OCH_2_O), 6.60 (d, *J*=8.2 Hz, 1H, 5′-H), 6.66 (d, *J*=8.2 Hz, 1H, 6′-H), 6.70 (s, 1H, 2′-H). ESI-MS *m/z* (%): 291.1 (M+Na^+^, 100).

### 3-(3, 4-Methylenedioxyphenyl)-2,2-dimethoxypropionic acid (12)

A 3.2 ml of KOH (aq., 5 N) and 3 ml of methanol were mixed together in a 25 ml flask. Compound **11** (180 mg, 0.67 mmol) was then added to the flask and the colour turned brownish red immediately. The pH was adjusted by adding 9% HCl (aq.) to 1–2 followed by extracted of EtOAc (6 ml×3) and dried over anhydrous Na_2_SO_4_. After filtration, the residue was evaporated to give compound **12** as a pale yellow solid (160 mg, 94%): mp 115–117°C. ^1^H NMR (400 MHz, CDCl_3_) δ 3.12 (s, 2H, –CH_2_–), 3.39 (s, 6H, (–OCH_3_)_2_), 5.93 (s, 2H, –OCH_2_O–), 6.63-6.73 (m, 3H, Ar–H). MS (EI) *m/z* (%): 254 (M^+^, 14.70), 209 (18.45), 149 (18.31), 135 (87.53), 119 (100.00), 105 (12.92), 77 (35.57), 59 (32.46). HRMS calcd mass for C_12_H_14_O_6_ 254.0790, found 250.0795.

### *N*-((*R*)-3-Benzylthio-1-methoxy-1-oxo-2-propanyl)-2,2-dimethoxy-3-(3,4 methylenedioxyphenyl)propanamide (8)

To a solution of carboxylic acid **12** (100 mg, 0.39 mmol) in CH_2_Cl_2_ (10 ml), methyl *S*-benzyl-L-cysteine hydrochloride (**13**) (103 mg, 0.39 mmol), HOBt (80 mg, 0.59 mmol), DIPEA (0.1 ml, 0.59 mmol) and EDC (113 mg, 0.59 mmol) were added successively. The mixture was stirred at room temperature for 4 h, and then purified by silica-gel column chromatography (eluent: PE-EtOAc, 3:1) to give the corresponding amide conjugate **8** as a white solid (127 mg, 70%): mp 56–58°C. ^1^H NMR (400 MHz, CDCl_3_) δ 2.63–2.65 (m, 2H, –CH_2_SBn), 3.09 (s, 2H, –CH_2_Ar), 3.33 (s, 3H, –OCH_3_), 3.38 (s, 3H, –OCH_3_), 3.57 (d, *J*=4.3Hz, 2H, –SCH_2_Ph), 3.68 (s, 3H, –COOCH_3_), 4.68-4.70 (m, 1H, –NHC***H***–), 5.81 (dd, *J*=11.5, 2.0 Hz, 2H, –OCH_2_O–), 6.62-6.67 (m, 3H, –Ar), 7.22–7.31 (m, 5H, –Ph). ESI-MS *m/z* (%): 484.4 (M+Na^+^, 100). HRMS calcd mass for C_23_H_27_NO_7_SNa [M+Na^+^] 484.1400, found 484.1406.

### Cell culture and treatment

Human neuroblastoma SH-SY5Y cells were obtained from Shanghai Institute of Material Medica, Chinese Academy of Sciences. Cells were cultured in DMEM supplemented with 10% NBS, 100 units/ml penicillin and 100 μg/ml streptomycin and kept at 37°C in a humidified 5% CO_2_ incubator. Cells were passaged every 2 days and always used at 80–90% cell confluence between passages 3 and 20. Cells were stored for 24 h before being exposed to various concentrations of Danshensu derivative (0.001, 0.01, 0.1, 1 and 10 μmol/l) or NAC (5 mmol/l) for 2 h followed by 150 μmol/l H_2_O_2_ for 24 h.

### Cell viability

Cell viability analysis was performed by MTT analysis [11]. Briefly, SH-SY5Ys were stored in a 96-well plate at a density of 0.3×10^4^ cells/well (DMEM with 10% NBS) and incubated overnight. After incubation, the medium was removed and replaced with a fresh medium without NBS (150 μl/well) and cells were pretreated with different concentrations of Danshensu derivatives and positive control NAC as described for 2 h prior subjected to 150 μmol/l H_2_O_2_ overnight. After that, 20 μl of MTT (5 mg/ml), which was dissolved in PBS solution was added to each well and the cells were incubated at 37°C for 4 h. Next, the medium was aspirated off, and 200 μl of DMSO was added to each well to achieve the coloured formazan in viable cells. The absorbance was read at 570 nm using a microplate reader (Tecan Infinite 200). Cell viability was presented as a percentage of the control cells.

### LDH measurement

The presence of cell damage can be observed by the release of LDH in the cultured medium. LDH is a cytoplasmic enzyme retained by viable cells with intact plasma membranes and released from cell with damaged membranes [12,13]. SH-SY5Y cells were incubated in 96 well plates with various concentrations (0.001, 0.01, 0.1, 1 and 10 μmol/l) of Danshensu derivatives for 2 h followed by 150 μmol/l H_2_O_2_ for 24 h. After treatment, the amount of LDH leakage by cells was evaluated using an assay kit according to the manufacturer's instruction. The absorbance was read at 490 nm.

### Western blotting

Cells were plated at 1.2×10^6^ cells/8 ml in 100-mm dish. After overnight treatment, cells were collected and washed with cold PBS. After centrifugation, cells were homogenized in 100 μl RIPA lysis buffer and incubated on ice for 20 min. Cell lysates were then centrifuged at 12000 ***g*** for 10 min at 4°C and the supernatant was collected. Protein concentration was determined using a BCA assay kit. A total of 50 μg of protein from each treatment condition were separated by SDS/PAGE and transferred to PVDF membranes (Millipore Corporation) using a Bio-Rad miniprotein-III wet transfer unit. Non-specific binding of antibody was blocked with 5% non-fat dried skimmed milk dissolved in TBST (pH 7.6, 10 mM Tris/HCl, 150 mM NaCl and 0.1% Tween-20) at room temperature for 1 h. Consequently, the membranes were washed three times and incubated with individual primary antibodies (Bax at 1:1000, Bcl-2 at 1:100, caspase 3 at 1:1000, SOD-2 at 1:100 and GAPDH at 1:1000) overnight at 4°C. After three times of washing with TBST buffer, the membranes were incubated with the anti-rabbit IgG secondary antibody (1:4000) in TBST containing 5% BSA for 1 h at room temperature followed by four times of washings. Signals were detected with chemiluminescence reagents (Beyotime) and quantified by densitometry using a Western blotting detection system (Alpha Innotech). The results were expressed as the percentage of control, which was deemed to be 100%.

### Nuclear staining with Hoechst 33258

The nuclear damage can be detected by staining the cells with Hoechst 33258. Hoechst 33258 is a blue fluorescent dye, which is sensitive to DNA conformation and chromatin state in cells. After treatment with or without Danshensu derivatives and induced death with H_2_O_2_ for 24 h, cells in 24-well plates were fixed and stained with DNA fluorochrome Hoechst 33258 for 20 min. After three times of washing with PBS, the morphology of the cells was monitored using a confocal microscope (LSM 510, ZEISS).

### MDA and GSH measurement

Cells were seeded to confluence in 24-well plates. After adding Danshensu derivatives for 2 h followed by 150 μmol/l H_2_O_2_ for 24 h, cells were harvested for the analysis. The cells were scraped from the plates into ice-cold RIPA analysis buffer (Beyotime). Protein concentration was then determined using the BCA protein assay kit. The measurement of MDA and GSH was conducted according to the manufacturer's protocol.

### Animal treatment and MCAO (middle cerebral artery occlusion)

The experimental protocol was approved by the ethical committee and confirmed to internationally accepted ethical standards. The animals were supplied by Laboratory Animal Centre, Fudan University. Hundred male SD (Sprague–Dawley) rats weighing 180-220 g were housed with food and water *ad libitum* under diurnal lighting condition. Animals were randomly divided into five groups: MCAO group with water treatment (Model); positive control group with Losartan (6.275 mg·kg^−1^·day^−1^) treatment; stroke groups were treated with 15, 30 and 60 mg·kg^−1^·day^−1^ of compound **6**. The drugs were given orally once daily for 7 days. After the pretreatment, the stroke was induced in the rats by MCAO as previously described [14]. Briefly, rats were anaesthetized with 7% trichloroacetaldehyde hydrate (C_2_H_3_Cl_3_O_2_) then, an incision of approx. 2 cm was made between the right orbit and the external auditory canal. A high-speed microdrill was used to make a small hole through the outer surface of the skull. After removing the dura, the right MCA was exposed and occluded using electro-cauterization from the point where it crossed the inferior cerebral vein to a point proximal to the origin of the lenticulostriate branches. To ensure the permanent occlusion, the branches of the MCA between these two points were occluded as well. Lastly, the wounds were later closed with sutures. Twenty-four hours afterthe surgery, rats were killed for sample collection.

### Infarct volume measurement

Twenty-four hours after MCAO, 10 rats from each group (model, sham, 15, 30 and 60 mg/kg), were killed. The brains were collected for the experiment; cerebellum and excess membranes were removed. The brains were sliced into 7–8 coronal pieces with 2 mm thickness each and stained with 0.1% TTC (2,3,5-triphenyltetrazolium chloride) solution at 37°C for 30 min and photographed. The infarct volume was measured as previous described [14].

### Measurement of SOD, GPx activity and MDA content

Twenty-four hours after MCAO, cortical tissues from six rats of each treatment group (model, sham, 15, 30 and 60 mg/kg) were collected and placed into an ice-cold RIPA lysis buffer. The samples were then homogenized using homogenizer and centrifuged at 10000 ***g*** for 5 min. Supernatant was collected for SOD, GPx and MDA measurements. The assays were performed according to the manufacturer's instruction.

### ROS (reactive oxygen species) production by DHE (**dihydroethidium)** staining

Twenty-four hours after MCAO, cortical tissues from two rats of each treatment group (model, sham, 15, 30 and 60 mg/kg) were collected, cut at the middle artery occluded area and then placed into the embedding medium [OCT (optimal cutting temperature)]. The samples were then placed in liquid nitrogen. For DHE staining, the samples were cut into 10 μm thickness and placed on the slides. After washing the sample slides three times with NPSS (normal physiological saline solution) (each wash of 5 min), the slides were incubated with NPSS containing 10 μM DHE at 37°C for 15 min avoiding the light. Following three times of washing with NPSS (each wash of 5 min), a drop of nail polish was added to the slide and covered with the cover slip. The ROS production was then monitored using a confocal microscope (LSM 710, ZEISS), fluorescence intensity measured at 515 nm for excitation and 585 nm for emission.

### Statistical analysis

Data were analysed by SPSS software and expressed as means±S.D. Two-tailed Students’ *t* test was carried out to verify statistical significance. Differences were considered significant at *P*<0.05.

## RESULTS

### Protective effects of Danshensu derivatives on H_2_O_2_-induced apoptosis in human neuroblastoma cells (SH-SY5Y)

**Figure 1:**
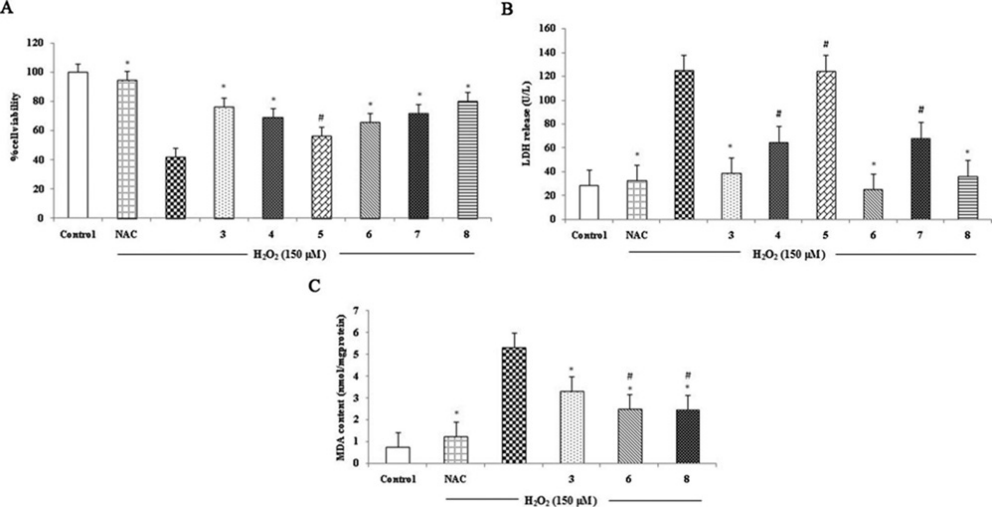
Effect of compounds **3**–**8** on MTT, LDH and MDA levels in H_2_O_2_-induced human neuroblastoma cells (SH-SY5Y) (**A**) Effects of compounds **3**–**8** at 1 μmol/l on cell viability in H_2_O_2_-induced human neuroblastoma cells (SH-SY5Y). (**B**) Effects of **3**–**8** at 1 μmol/l on LDH release in H_2_O_2_-induced human neuroblastoma cell (SH-SY5Y). (**C**) Effects of compounds **3**, **6** and **8** at 1 μmol/l on the MDA level in H_2_O_2_-induced human neuroblastoma cells (SH-SY5Y). Values are expressed as means±S.D. from six individual samples. *P<0.05 against the H_2_O_2_-treated group, #*P*<0.05 against compound **3**.

All compounds were evaluated for their effects on cell viability of H_2_O_2_-induced apoptosis in SH-SY5Y. Cells were incubated with 1 μmol/l Danshensu conjugate derivatives (**3–8**) for 2 h before exposure to 150 μmol/l H_2_O_2_ for 24 h. MTT analysis was performed to evaluate cell viability and NAC was used as positive control in the experiment. Exposure to H_2_O_2_ reduced cell viability to 40–50%; however, pretreatment with all compounds except compound **5**, significantly increased cell viability and is similar to the effect of NAC (5 mmol/l) (*P<0.05 against the H_2_O_2_-treated group, #*P*<0.05 against compound **3**) (Figure 1A). LDH leakage result was coincident with the MTT assay, but compounds **3**, **6** and **8** showed statistical significance (Figure 1B).

**Figure 2:**
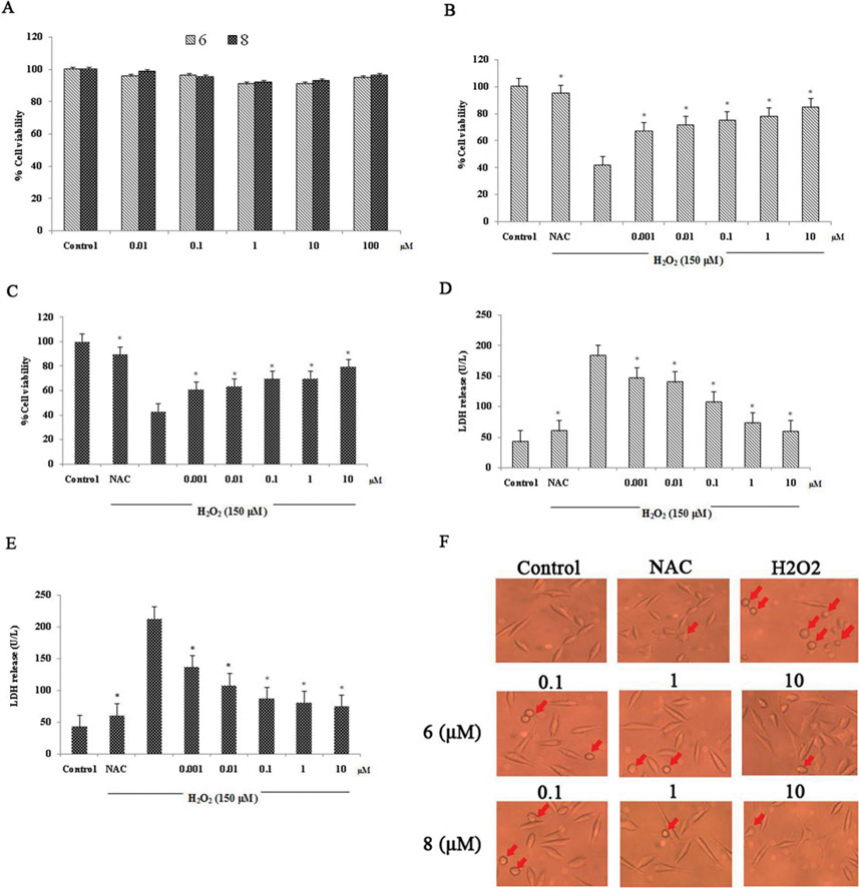
Effect of compounds **6** and **8** on MTT, LDH and morphological alteration at different concentrations in H_2_O_2_-induced human neuroblastoma cells (SH-SY5Y) (**A**) Effects of various concentrations of compounds **6** and **8** alone on cell viability. (**B**) Effect of various concentrations of compound **6** (0.001, 0.01, 0.1, 1 and 10 μmol/l) on cell viability in H_2_O_2_-induced SH-SY5Y. (**C**) Effect of various concentrations of compound **8** (0.001, 0.01, 0.1, 1 and 10 μmol/l) on cell viability in H_2_O_2_-induced SH-SY5Y. (**D**) Effect of various concentrations of compound **6** (0.001, 0.01, 0.1, 1 and 10 μmol/l) on LDH release in H_2_O_2_-induced SH-SY5Y. (**E**) Effect of various concentrations of compound **8** (0.001, 0.01, 0.1, 1 and 10 μmol/l) on LDH release in H_2_O_2_-induced SH-SY5Y. (**F**) Effects of compounds **6** and **8** on H_2_O_2_-induced morphological alterations in SH-SY5Y (×400). Values are expressed as means±S.D. from six individual samples.*P<0.05 against the H_2_O_2_ treated group.

According to the preliminary results of protective effect of Danshensu derivatives, two Danshensu-cysteine derivatives including compounds **6** and **8** were chosen for further evaluation. The protective effect at different concentrations of compounds **6** and **8** (0.001, 0.01, 0.1, 1 and 10 μmol/l) was evaluated by MTT and LDH assays. NAC served as the positive control in the experiment. The concentrations of compounds **6** and **8**, which were used in the experiment alone did not affect cell viability (Figure 2A). After incubation with various concentrations of compounds **6** and **8** for 2 h, cells were exposed to 150 μmol/l H_2_O_2_ for 24 h. As shown in Figures 2(B) and 2(C), pretreatment with the indicated concentrations of compounds **6** and **8** could significantly restore cell viability from 40–50% to 70–90%.

In LDH assay, increased level of LDH leakage in H_2_O_2_-treated cells was reduced by different concentrations of both compounds **6** and **8** (Figures 2D and 2E) (*P<0.05 against the H_2_O_2_-treated group).

### Anti-apoptotic activity of compounds 6 and 8 on H_2_O_2_-induced apoptosis in SH-SY5Y cells

**Figure 3:**
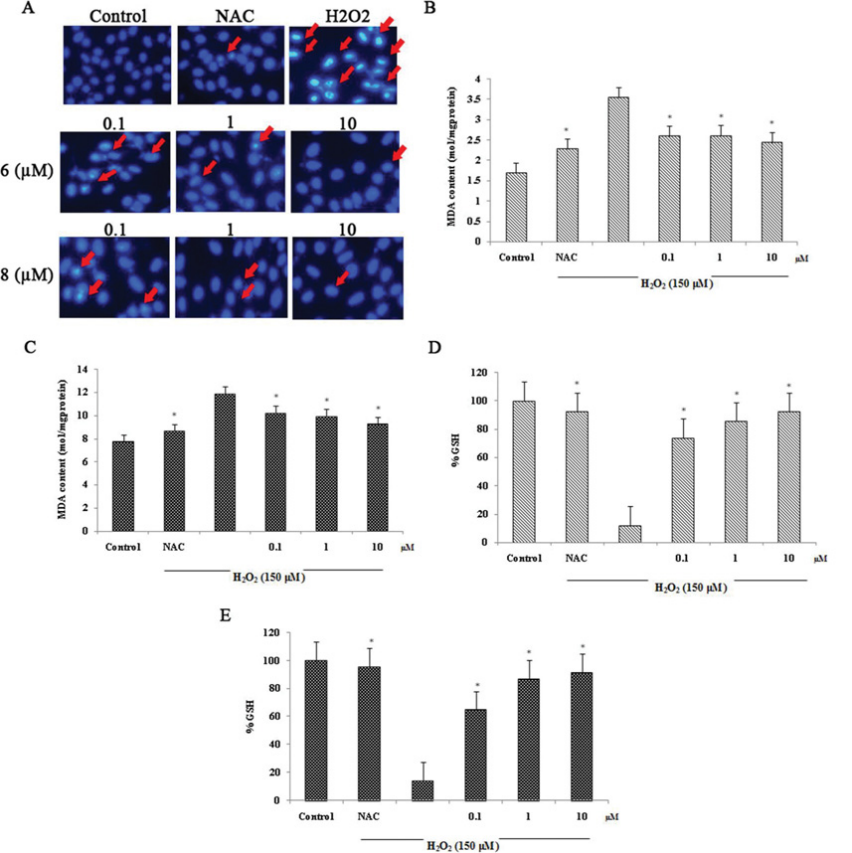
Effect of compounds **6** and **8** on Hoechst staining, MDA and GSH levels at different concentrations in H_2_O_2_-induced human neuroblastoma cells (SH-SY5Y) (**A**) Effects of compounds **6** and **8** on H_2_O_2_-induced morphological alteration in SH-SY5Y. Fluorescence photomicrographs of nuclear stained with Hoechst 33258 (×400). (**B**) Effect of compound **6** on MDA content in H_2_O_2_-induced SH-SY5Y. (**C**) Effect of compound **8** on MDA content in H_2_O_2_-induced SH-SY5Y. (**D**) Effect of compound **6** on GSH level in H_2_O_2_-induced SH-SY5Y. (**E**) Effect of compound **6** on GSH level in H_2_O_2_-induced SH-SY5Y. Values are expressed as means±S.D. from three individual samples. *P<0.05 against the H_2_O_2_ treated group.

Hoechst staining was performed to observe morphological changes. Apoptotic cell is characterized by cell shrinkage, nuclear condensation and DNA fragmentation. Cells treated with compounds **6** and **8** at a range of concentration (0.1, 1 and 10 μmol/l) and NAC (5 mmol/l) prior to H_2_O_2_ exposure indicated less nuclear shrinkage and condensation compared with the H_2_O_2_-treated cell group (Figure 3A) (*P<0.05 against the H_2_O_2_ treated group).

### Antioxidant activity of compounds 3, 6 and 8 on H_2_O_2_-induced apoptosis in SH-SY5Y cells

MDA, a marker of oxidant-mediated lipid peroxidation in cells was measured after H_2_O_2_ treatment. Treatment with H_2_O_2_ resulted in significant increase in the MDA level. Pretreatment with NAC (5 mmol/l), compounds **3**, **6** and **8** at 1 μmol/l for 2 h prior to H_2_O_2_ incubation for 24 h could significantly inhibit the increase in the MDA level. However, compounds **6** and **8** indicated to be more effective than compound **3** (Figure 1C) (*P<0.05 against the H_2_O_2_-treated group, #*P*<0.05 against compound **3**).

Compounds **6** and **8** at a range of concentration (0.1, 1 and 10 μmol/l) and NAC (5 mmol/l) were then observed the activity on the level of MDA and GSH, the major endogenous antioxidant produced by cells. Treatment with H_2_O_2_ results in significant increase in the MDA level, while a decrease in the GSH level. Pretreatment with NAC, compounds **6** (Figures 3B and 3D) and **8** (Figures 3C and 3E) for 2 h prior to H_2_O_2_ incubation for 24 h could inhibit the increase in the MDA level and enhance the level of GSH in a concentration-dependent manner.

### Effect of compounds 6 and 8 on the apoptosis marker proteins Bcl-2, Bax and caspase 3 and antioxidant protein SOD-2 on H_2_O_2_-induced apoptosis in SH-SY5Y cells

**Figure 4:**
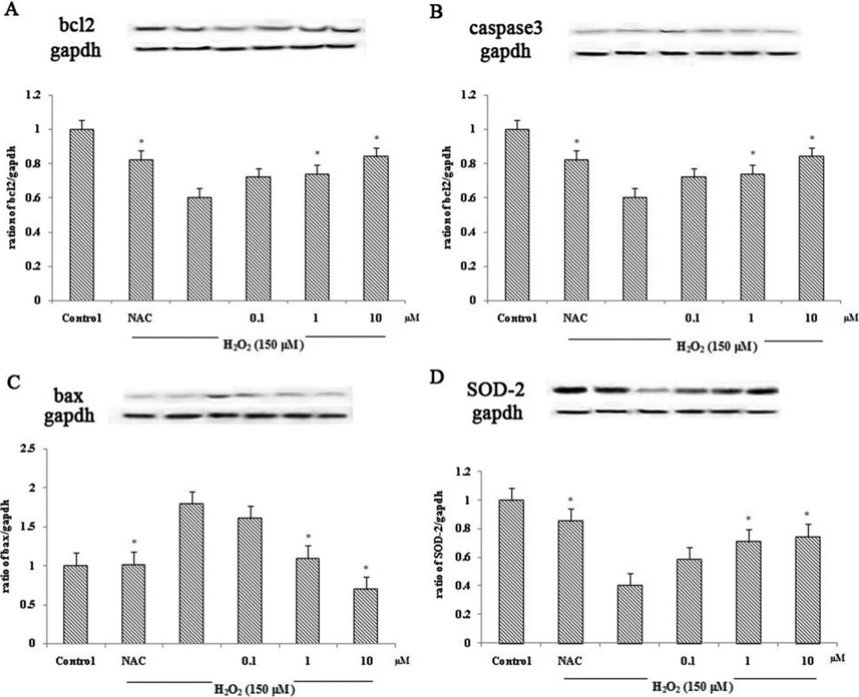
Effect of compounds **6** on protein expression (**A**) Effect of compound **6** on the expression of Bcl-2 protein in H_2_O_2_-induced SH-SY5Y. (**B**) Effect of compound **6** on the expression of caspase 3 protein in H_2_O_2_-induced SH-SY5Y. (**C**) Effect of compound **6** on the expression of Bax protein in H_2_O_2_-induced SH-SY5Y. (**D**) Effect of compound **6** on the expression of SOD-2 protein in H_2_O_2_-induced SH-SY5Y. Values are expressed as means±S.D. from three individual samples. *P<0.05 against the H_2_O_2_ treated group.

**Figure 5:**
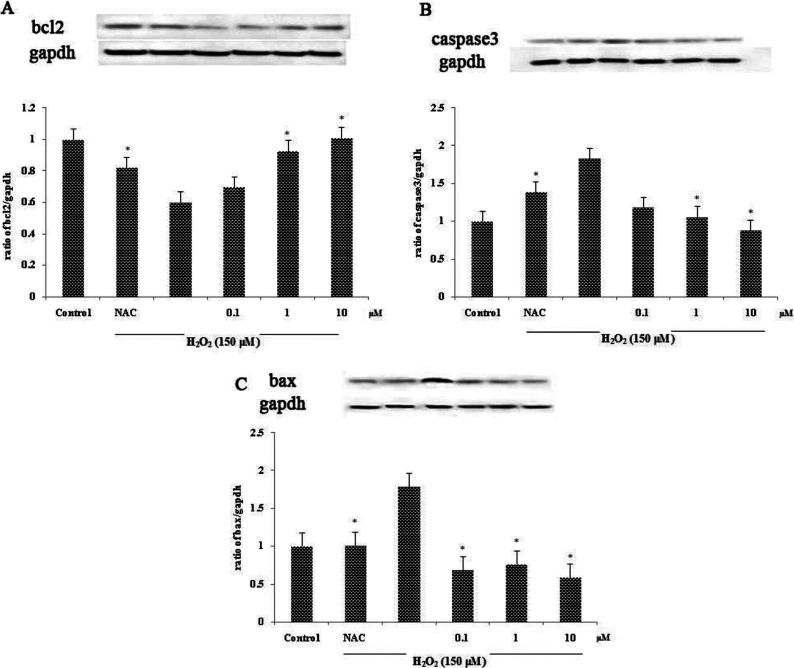
Effect of compound **8** on protein expression (**A**) Effect of compound **8** on the expression of Bcl-2 protein in H_2_O_2_-induced SH-SY5Y. (**B**) Effect of compound **8** on the expression of caspase 3 protein in H_2_O_2_-induced SH-SY5Y. (**C**) Effect of compound **8** on the expression of Bax protein in H_2_O_2_-induced SH-SY5Y. Values are expressed as means±S.D. from three individual samples. *P<0.05 versus the H_2_O_2_ treated group.

To observe the expression of apoptotic protein, we analysed the expression of Bcl-2, Bax and caspase 3 proteins in cell lysates using Western blotting. GAPDH served as the internal standard. Cells were treated with or without compounds **6** and **8** for 2 h prior to 24 h of H_2_O_2_ exposure. An expression of Bcl-2 protein was significantly decreased in the H_2_O_2_ treated group. However, pretreatment with compounds **6** and **8** could restore the Bcl-2 protein level (Figure 4A and 5A). In contrast, the high level of caspase 3 protein observed in the H_2_O_2_ treated group was decreased dose dependently in the pretreated group (Figures 4B and 5B). The result of the Bax protein level was consistent with those of the caspase 3 protein level (Figures 4C and 5C). The effect of compound **6** on the expression of antioxidant protein SOD-2 was also observed. Decrease in the level of SOD-2 protein expression in the H_2_O_2_ treated group could be restored by pretreatment with compound **6** in a dose-dependent manner (Figure 4D).

### *In vivo* protective effect of compound 6 on acute cerebral ischaemia in adult rats

**Figure 6:**
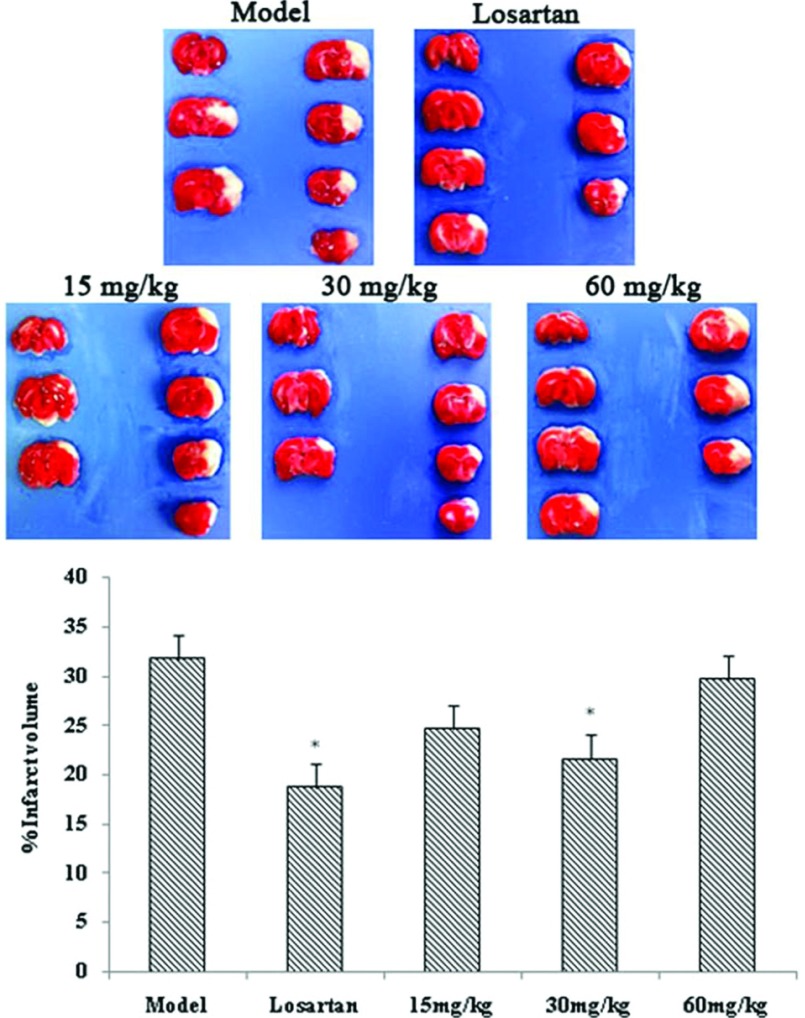
Effect of compound 6 at different concentrations on infarct volume in rat after cerebral infarction Values are expressed as means±S.D. from ten individual samples. *P<0.05 versus the model group.

**Figure 7:**
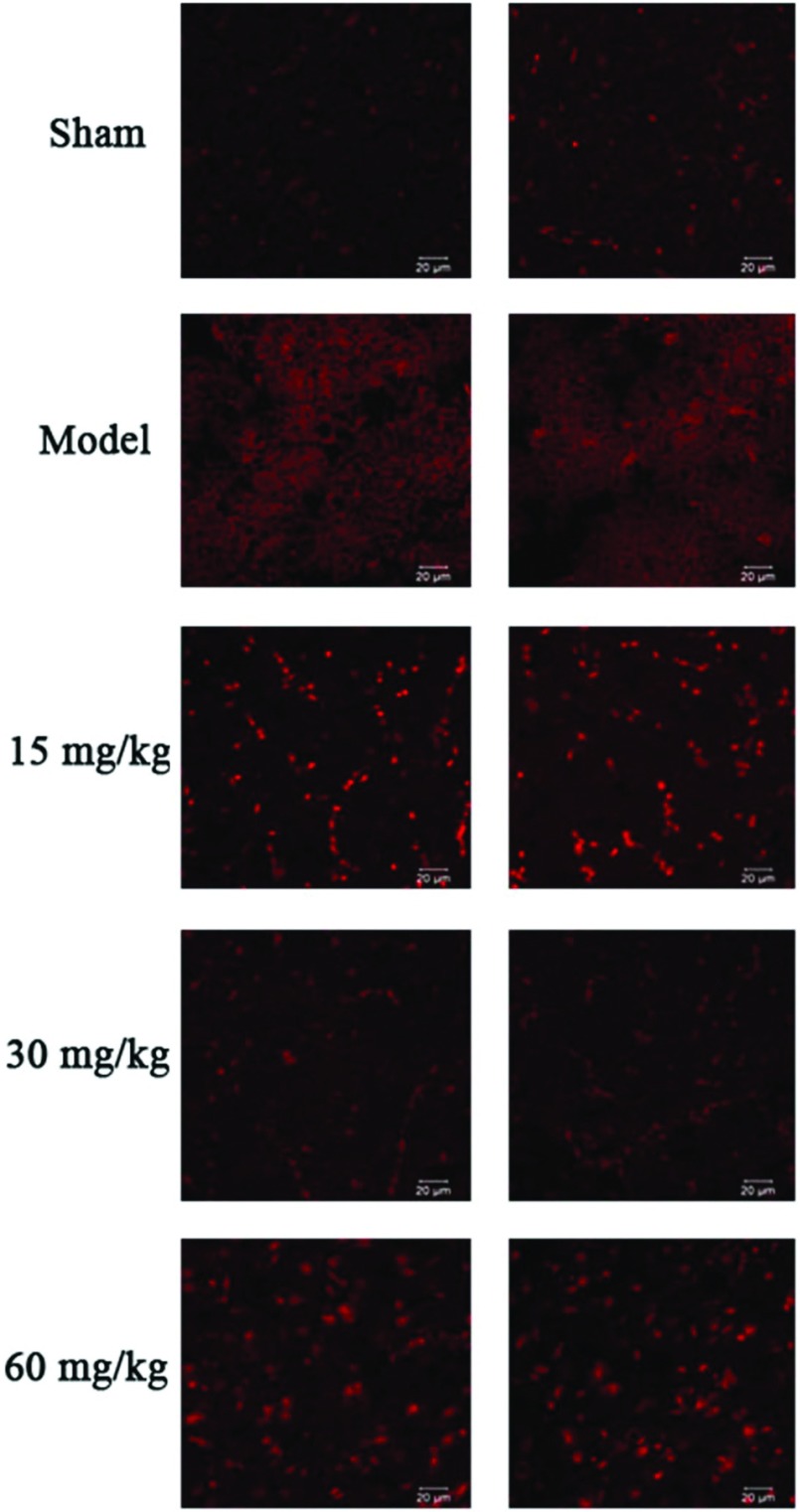
Effect of compound 6 on ROS production assessed by DHE staining

The effect of compound **6** was further studied in an animal model due to the potent protective effect indicated in an *in vitro* study. MCAO was used as a model of acute cerebral ischaemia. Rats were orally treated with compound **6** at different concentrations (15, 30 and 60 mg/kg) daily for 7 days. Twenty-four hours after MCAO, the brains were collected and the infarct size was measured using 0.1% TTC staining. Pre-incubation with compound **6** reduced the infarct area compared with the model rats and at 30 mg/kg it is statistically significant (Figure 6). The level of clinical markers for oxidative stress was also evaluated. Insulted rats with permanent MCAO demonstrated the significant reduction in activities of SOD, GPx and increase in the MDA level. Rats treated with compound **6** could increase the activities of SOD, GPx and decrease the level of MDA notably (Table. 1). Moreover, MCAO insulted rat demonstrated the increase in ROS production, and which could be effectively eliminated with compound **6** treatment (Figure 7).

**Table 1:** Effects of compound 6 on MDA level and SOD and GPx activity in rat after cerebral infarction Values are expressed as means±S.D. from six individual samples. *P<0.05 versus model group, #*P*<0.05 versus sham group.

	SOD (units/mg)	GPx (units/mg)	MDA (nmol·l^−1^·mg^−1^)
Sham	20.11±7.21	99.42±16.28	1.51±1.36
Model	6.19±2.14#	43.56±14.69#	6.46±1.55#
60 mg/kg	12.71±4.19*	58.21±20.34*	4.42±1.47*
30 mg/kg	17.07±4.42*	71.51±12.85*	3.55±1.17*
15 mg/kg	13.47±1.22*	65.49±18.32*	3.94±2.15*

## DISCUSSION

The current study was performed to evaluate the pharmacological activity of Danshensu derivatives on H_2_O_2_-induced human neuroblastoma (SH-SY5Y) injury. In the experiment, H_2_O_2_ was used for ischaemia induction as it is extensively used as a stimulus to induce apoptosis [15–17]. The study represents that Danshensu derivatives (**3**, **4**, **6**, **7** and **8**) exhibited a significant protective effect on H_2_O_2_-induced neuronal cell (SH-SY5Y) injury. For Danshensu derivative compound **3**, the results were consistent with the previous study, which showed its protective activities against hypoxia-induced myocardial cell damage [10]. For the cysteine conjugates, it seems to be well-ordered structure–activity relationship. The amide conjugates **4** and **6** were privileged than thioester conjugate **7**. The results were consistent with our previous study, it has shown that the phenolic acetyl protective groups and 2-acetoxy substitution in amide conjugates **4** and **6** are favourable to pharmacological activity [1]. In the case of the amide conjugate **5**, it showed the reverse effect when compared with others, this may be described by the fact that their methylene phenolic protective groups were difficult to cleave to release hydroxyls compared with acetyl in compounds **4** and **6**. For the amide conjugate **8**, although it has got the methylene phenolic protective groups as well as compound **5**, the results showed that compound **8** possessed effects similar to compound **6**. This may suggest that the methylenedioxyphenyl could facilitate its activity. Comparing the cysteine compounds with compound **3**, the results of MTT, LDH leakage showed that compounds **6** and **8** had effects similar to compound **3** (Figures 1A and 1B). However, from the result of MDA content, compounds **6** and **8** showed more effects than compound **3** (Figure 1C). This may suggest that compound **3** possessed less effect than compounds **6** and **8** in antioxidant property. Thus, at the same concentration Danshensu-cysteine derivative compounds **6** and **8** were found to be more effective than others. Further study of compounds **6** and **8** indicated that they exert the potent neuroprotective effect in a dose-dependent manner on the cellular ischaemic stroke model induced by H_2_O_2_. Compound **6** also showed the anti-ischaemic effect in animal study.

The protective effect of the compounds in an *in vitro* study was determined using MTT viability assay and LDH release. Decreased cell viability in MTT assay and increased LDH release were observed in SH-SY5Y cells which were exposed to H_2_O_2_. Treatment with Danshensu-cysteine derivatives (**6** and **8**) prior to H_2_O_2_ treatment significantly increased the number of survival cells and prevented LDH leakage in a dose-dependent manner. As a positive control NAC was used as it is an antioxidant drug and helps to protect cell death [18]. These data correspond to the morphological changes of SH-SY5Y cells observed under phase contrast microscopy. Apoptosis is known to participate in many biological processes such as maintenance and development of tissue homoeostasis. Dysfunction of apoptosis has been implicated in various forms of human diseases including neurodegenerative diseases and ischaemic stroke [19–20]. Previous research in our group demonstrated that compound **6** has anti-apoptosis in CVD (cardiovascular disease) [9]. In the present study, the anti-apoptotic capacity of Danshensu-cysteine derivatives in neurodegenerative disease was also evaluated. In order to assess the effectiveness of the individual compounds, apoptotic proteins were measured by means of Western blotting. The apoptotic proteins including Bcl-2, Bax and caspase 3 were measured. Bcl-2, an anti-apoptotic protein, functions by forming heteromers with apoptotic Bcl-2 members to sequester their anti-apoptotic function [21–22]. Bax, a pro-apoptotic protein, induces cell death by targeting outer mitochondrial membrane [23]. Caspase 3, an enzyme that plays a major role in apoptosis, is activated in the apoptotic cell both by extrinsic and intrinsic apoptotic pathways [24]. The expression of Bcl-2 in SH-SY5Y cells that were subjected to H_2_O_2_ markedly decreased while the protein expression of Bax and caspase 3 significantly increased. Treatment with compounds **6** and **8** before exposure to H_2_O_2_ could significantly up-regulate Bcl-2 protein expression and down-regulated Bax and caspase 3 protein expression in a concentration-dependent manner. Further, the apoptosis can be observed by staining the cells with a blue florescence dye which is sensitive to DNA conformation and chromatin state within a cell (Hoechst staining). Treatment with H_2_O_2_ results in cell death; use of these derivatives displayed a marked reduction in cell death in a dose-dependent manner.

Oxidative stress is one of the two important pathophysiological events involved in ischaemic stroke. It occurs when there is an imbalance between oxidant and anti-oxidant [25]. MDA is a by-product of oxidation of poly-unsaturated fatty acid; therefore, it is used as a marker of oxidative stress. GSH is the major free radical scavenger in the brain; reduction of GSH level promotes cellular vulnerability towards oxidative stress. GSH neutralizes the free radicals and reactive oxygen compounds and maintains exogenous antioxidants in their active forms [26,27]. The level of MDA in SH-SY5Y cells that were exposed to H_2_O_2_ increased significantly while the level of GSH markedly decreased. The addition of compounds **6** and **8** notably reduced the level of MDA and restored the level of GSH. SOD-2 or manganese SOD is a member of SOD family which is the enzyme that converts superoxide radicals in mitochondria into less toxic agents [28]. Treatment with compound **6** could up-regulate the SOD-2 expression. This indicated that compounds **6** and **8** exerted an antioxidation effect through inhibiting the by-product of oxidative stress and promoting the activity of free radical scavenger and the expression of enzyme that works on converting radical into less toxic agents. *In vivo* study consistently demonstrated that the right MCAO caused the infarction in the right cortical area and corpus striatum shown by TTC staining. Treatment with compound **6** at 30 mg·kg^−1^·day^−1^ could markedly reduce the infarct volume as compared with the model group. To investigate the possible mechanism, the antioxidant activity was evaluated by observing the changes in endogenous antioxidant enzyme activities. SOD converts superoxide radicals into less toxic agents. GPx scavenges and inactivates hydrogen and lipid peroxides, thereby protecting the body against oxidative stress [28]. MDA was also measured as it is one of the biomarkers for oxidative stress as described above. The present work tested the antioxidant properties in the MCAO rat model. The indication of severe oxidative stress during cerebral ischaemia appeared *via* the increase in the number of free radicals and the decrease in endogenous antioxidants. The decrease in the activity of SOD and GPx and increase in the MDA level in the rat model group suffered from permanent MCAO indicated that the severe oxidative stress takes place during the permanent MCAO. With compound **6** at 15 and 30 mg·kg^−1^·day^−1^ treatment, an increase in the activity of SOD and GPx and a decrease in the MDA level were observed. Moreover, to directly demonstrate the anti-oxidative stress capacity, the ROS production was also observed using DHE staining. DHE has been widely used for tissue experiments to determine ROS production as it can freely permeate through cell membranes [29–31]. The reaction of DHE and superoxide anions, resulted in a red fluorescent product (ethidium), which is intercalated with DNA [32–33]. MCAO insulted rat demonstrated an increase in ROS production and that could be eliminated with the treatment of compound **6**. According to the observed underlying mechanism, the protective effect of compound **6** may also involve in the antioxidation pathway. However, the therapeutic window for the optimal dosage of compound **6** may have a very narrow range from 15 to less than 60 mg·kg^−1^·day^−1^ according to the infarct volume, ROS production, the activity of SOD, GPx and MDA levels. The results showed that treatment with compound **6** at 15 and 30 mg·kg^−1^·day^−1^ reduced the infarct volume, decreased ROS production and MDA level and also enhanced the activity of SOD and GPx. However, treatment at 60 mg·kg^−1^·day^−1^ showed less effect than treatment at 15 and 30 mg·kg^−1^·day^−1^. This may suggest that compound **6** at 60 mg·kg^−1^·day^−1^ is toxic.

### Conclusion

Pharmacological evaluation of several novel amide and thioester conjugates between Danshensu and L-cysteine derivatives (**4**–**8**) and Danshensu derivative with the phenolic hydroxyls esterized (**3**) have been reported. In this study, we have shown significant neuroprotective effects of compounds **3**, **4**, **6**, **7** and **8** on human neuroblastoma cells under H_2_O_2_-induced cell apoptosis. Their protective effects may involve anti-apoptosis and anti-oxidative stress. Compound **6** also exhibit an anti-ischaemic effect by blocking the oxidative stress and apoptotic pathway in MCAO insulted rats. With regard to these promising pharmacological results, compound **6** merits the potential anti-cerebral ischaemic drug candidate and further study of this mechanism is needed.
